# Value of MRI derived parameters in the discrimination of familial left ventricular noncompaction (LVNC), DCM and HCM in comparison to healthy volunteers

**DOI:** 10.1186/1532-429X-13-S1-P269

**Published:** 2011-02-02

**Authors:** Matthias Gutberlet, Milena Pachowsky, Matthias Grothoff, Janine Hoffmann, Christian Lücke, Sabine Klaasen, Maximilian Posch, Sabine Hassfeld

**Affiliations:** 1 Heart Center Leipzig-Radiology, Leipzig, Germany; 2 Max-Delbrück- Center for Molecular Medicine Berlin, Berlin, Germany; 3 Charité, Campus Berlin-Buch/Franz-Volhard-Klinik, Berlin, Germany

## Objective

To evaluate the value of different MRI derived parameters to differentiate left ventricular noncompaction of the myocardium (LVNC) in patients with known familial LVNC from other cardiomyopathies.

## Background

LVNC is a rare disease characterized by numerous, excessively prominent ventricular trabeculations and deep intertrabecular recesses communicating with the ventricular cavity. Cardiac MRI (cMRI) has been reported as an ideal imaging modality to characterize patients with LVNC. Nevertheless, qualitative parameters to differentiate normal compaction of the myocardium in healthy subjects from LVNC or from other cardiomyopathies like dilated cardiomyopathy (DCM) or hypertrophic cardiomyopathy (HCM) may fail due to highly variable left ventricular trabeculations. Therefore, absolute quantification should be performed.

## Material and methods

**Figure 1:**
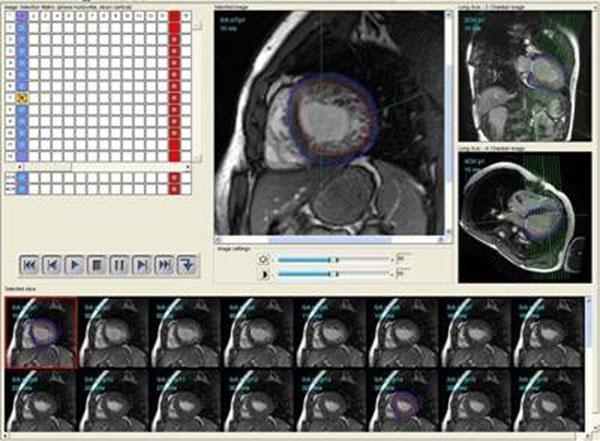


We examined 11 morphologically affected patients (34.8 years±18) with known familial LVNC carrying a gene mutation, 11 DCM (32.6 years±17) and 10 HCM patients compared with 24 healthy volunteers (20.2 years±6.3) using a 1.5 T scanner and a standard steady state free precession sequence in standard orientations. Total left ventricular muscle mass (MM), the compacted muscle mass (MM_comp_), MM-Index=MMI [g/m^2^], MM_non-comp_, percental MM_non-comp_, ventricular volumes and function were calculated using the CAAS MRV (Fig.1) software(Pie Medical Imaging, Maastricht, Netherlands). Furthermore, inversion recovery gradient echo sequences (IR-GRE) were performed 10-20 min. after the administration of 0.2 mmol/kg body weight Gd-DTPA i.v. (Magnevist, BayerSchering, Berlin, Germany). Additionally, a semi-quantitative segmental analysis according to the 17-segment model of the AHA of the occurrence of increased trabeculations and a ROC-analysis was performed.

## Results

**Figure 2:**
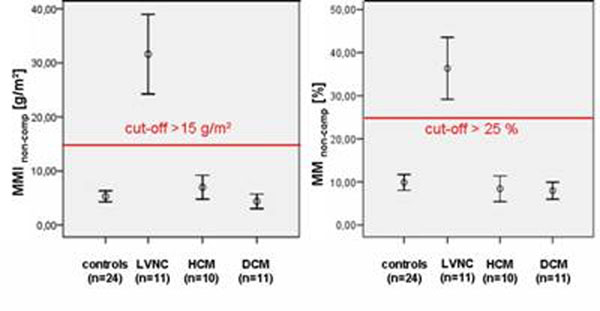


The mean EF was significantly reduced (p<0.05) in LVNC patients compared to all cardiomyopathies and healthy subjects, total MM was increased, but not significantly different from HCM patients. The total MMI_non-comp_ and percental MM_non-comp_ were good discriminators between healthy subjects and other cardiomyopathies with cut-offs of 15 g/m^2^ and 25%, respectively (Fig. 2). Sensitivities and specificities of 91/100 and 91/98 could be achieved. The echocardiographical criteria of the MM_non-comp_ /MM_comp_-ratio>2/1 in at least one segment demonstrated a very good sensitivity (100%), but only a poor specificity (27%). None of the LVNC patients demonstrated with intramyocardial late gadolinium enhancement (LGE), but HCM and DCM patients did. Furthermore, increased trabeculations in the basal and septal segments, especially in the segments 4-6, are additional strong indicators for LVNC.

## Conclusion

Absolute cMRI quantification of the MMI_non-comp_ or the percental MM_non-comp_ together with an absence of LGE and increased trabeculations in basal segments allows to reliably diagnose familial LVNC.

